# Transmyocardial therapeutic-delivery using real-time MRI guidance

**DOI:** 10.1186/1532-429X-15-S1-P15

**Published:** 2013-01-30

**Authors:** Shashank S Hegde, Steven Shea, Li Pan, Parag Karmarkar, Julien Barbot, Klaus J Kirchberg, Fijoy Vadakkumpadan, Jeremy Maurer, Judy A Cook, Natalia Trayanova, Meiyappan Solaiyappan, Peter V Johnston, Dara Kraitchman

**Affiliations:** 1 Radiology, Johns Hopkins University, Baltimore, MD, USA; 2 Biomedical Engineering, Johns Hopkins University, Baltimore, MD, USA; 3 Center for Applied Medical Imaging, Siemens Corporation, Corporate Research and Technology, Baltimore, MD, USA; 4 MRI Interventions, Irvine, CA, USA; 5 Department of Medicine, Division of Cardiology, Johns Hopkins University, Baltimore, MD, USA; 6 Center for Applied Medical Imaging, Siemens Corporation, Corporate Research and Technology, Princeton, NJ, USA

## Background

Catheter-based transmyocardial injection offers a minimally invasive method to deliver therapeutics to the heart. It is typically performed under X-ray fluoroscopic guidance, which suffers from poor demarcation of myocardial boundaries and an inability to assess myocardial viability. MRI-guided intramyocardial delivery of therapeutics at 3T offers the potential for more precise targeting of these therapies with superior tissue contrast. Our group has been actively involved with microencapsulated stem cell therapy to improve cell retention and prevent stem cell rejection. However, conventional microencapsulated stem cell products are too large to be administered transmyocardially. We demonstrate here intramyocardial injection of a prototype single stem cell therapeutic into the myocardium of a normal swine using real-time MR guidance and a custom active injection catheter.

## Methods

**Figure 1:**
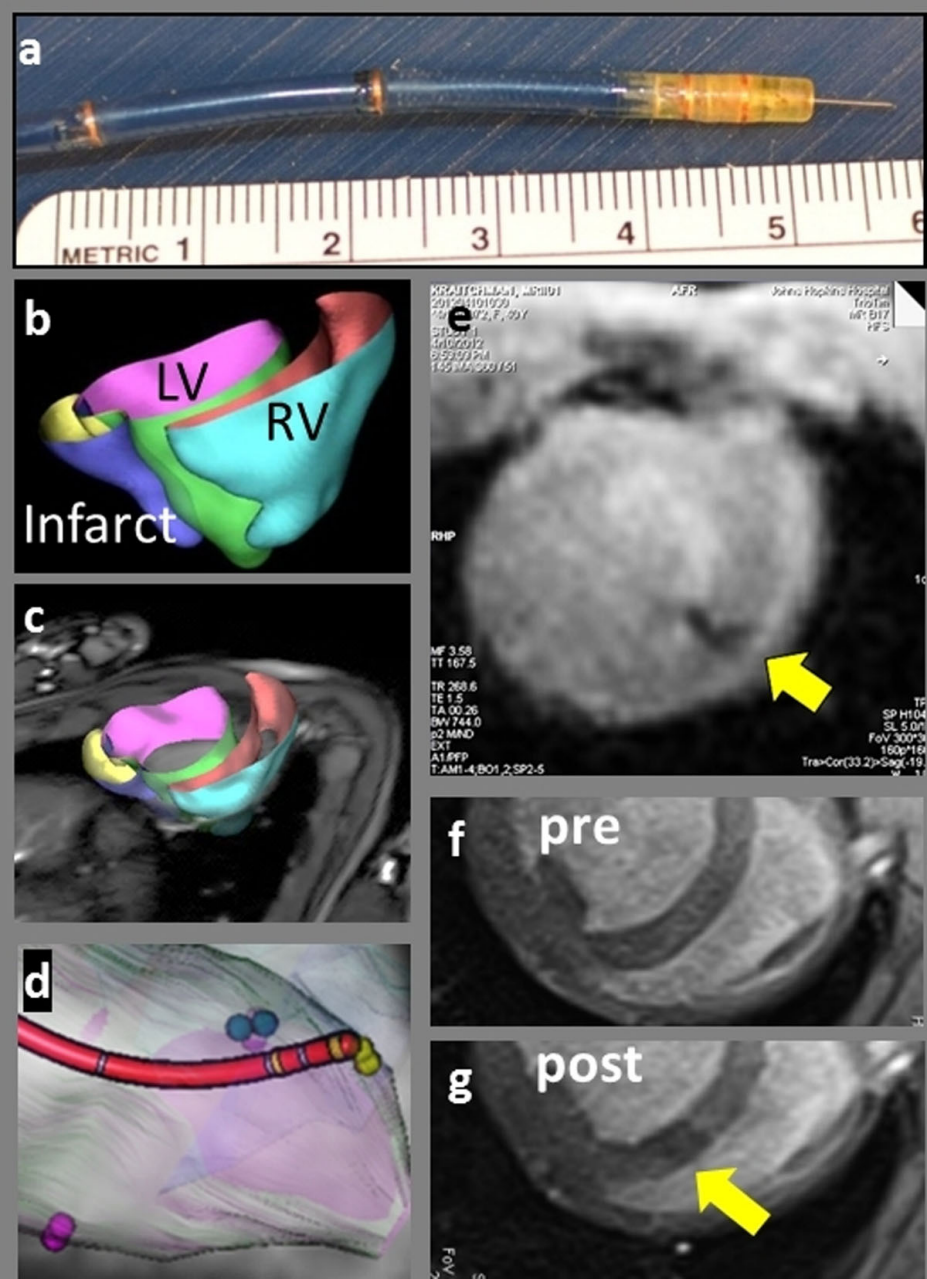
(a) Needle catheter showing microcoils and extended nitinol injection needle. (b) Portions of the heart segmented on cine stack (TR/TE=3.4/1.54 ms, voxel=1.3x1.3x5 mm-cube, FA 43 degrees) are used to build 3d model of the left, right ventricle and infarcted region. (c) 3D model overlaid into real-time MR images is used to (d) navigate the catheter to pre-defined injection targets. Catheter model is built from MR images of active tracking coils. (e) Injection of iron-oxide laden therapeutic into myocardium (yellow arrow) monitored under real-time MRI. TR/TE=2.8/1.19 ms, voxel=1.9x1.9x5 mm^3^, FA/tracking FA =50/15 degrees, 1.5-4 frames/s. (f, g) Pre- and post-injection MRI confirms injection of microbeads. TR /TE = 6.8/3.25 ms, FA=30 degrees, voxel =1.0×1.0×5.0 mm-cube.

Prototype alginate microbeads (50 µm diameter) impregnated with iron oxide (MyeOne) were produced using a custom microfluidic device. A custom MR- trackable, steerable intramyocardial injection catheter (10F diameter, 135 cm long, MRI Interventions, Inc.) with four built-in tracking coils (Figure 1a) was visualized on the Interactive Front End (IFE) navigation software (Siemens Corporate Research) in conjunction with a real-time tip-tracking sequence (BEAT_IRTTT, Pan.L et.al, ISMRM 2011, pp. 195). The catheter and the real-time interface were tested in a phantom, *ex vivo* pig heart, and an in vivo swine study at 3T (Siemens TimTrio). For the in vivo study, a breath-hold, multi-slice cine TrueFISP short-axis stack was obtained, which was segmented and converted to a 3D model (Figure 1b) for an overlay within IFE (Figure 1c). The catheter was navigated using real-time model guidance to target injection sites (Figure 1d). After ensuring correct needle placement, iron oxide-impregnated alginate microbeads (0.02 ml/injection) were injected into the myocardium during a FLASH real-time acquisition (Figure 1e). Delivery success was confirmed using a breath hold, multi-slice 2D FLASH sequence (Figure 1f,g). Relevant imaging parameters are in the figure caption.

## Results

Left ventricular catheterization and guidance to four target sites in the myocardium was achieved. Confirmation of microbead delivery on FLASH images was possible but difficult due to the small volume and low iron concentration delivered. Follow-up imaging at one week post-delivery was unable to visualize the iron oxide-labeled therapeutic. No adverse events from transmyocardial delivery were observed.

## Conclusions

A real-time interface with active catheter tip tracking enabled successful 3T MRI-guided transmyocardial delivery of a prototype therapeutic to the *in vivo* heart.

## Funding

MD Stem Cell Research Foundation (2011-MSCRFII-0043) and Siemens Corporation

